# Antimicrobial susceptibility profiles of *Escherichia coli* and *Klebsiella pneumoniae* isolated from outpatients in urban and rural districts of Uganda

**DOI:** 10.1186/s13104-016-2049-8

**Published:** 2016-04-25

**Authors:** Christine F. Najjuka, David P. Kateete, Henry M. Kajumbula, Moses L. Joloba, Sabiha Y. Essack

**Affiliations:** Department of Medical Microbiology, School of Biomedical Sciences, College of Health Sciences, Makerere University, Kampala, Uganda; Department of Immunology and Molecular Biology, College of Health Sciences, Makerere University, Kampala, Uganda; Antimicrobial Research Unit, School of Health Sciences, University of KwaZulu-Natal, Westville, Durban, South Africa

**Keywords:** Antimicrobial resistance, *Escherichia coli*, *Klebsiella pneumoniae*, Outpatient clinic, Commensal bacteria, Urban, Rural, Kampala, Uganda

## Abstract

**Background:**

Antimicrobial resistance is a global public health concern contributing to increased morbidity and mortality particularly in low-income countries. Studies on commensal bacteria are important as they reflect the state of antimicrobial susceptibility patterns in populations. However, susceptibility data on potentially pathogenic commensal bacteria from individuals in communities are still limited. The aim of this cross-sectional study was to determine the susceptibility profiles of *Escherichia coli* and Klebsiella species isolated from clients attending outpatient clinics in Kampala (urban district) and two rural districts of Uganda, Kayunga and Mpigi. Factors associated with such carriage are also reported.

**Results:**

A total of 1448 participants were recruited into the study with 985 yielding organisms of interest from stool or urine samples (one per client). Most growth occurred from stool samples (636/985, 87 %), of which 620/636 (97 %) grew *E. coli* while 16 (3 %) were *Klebsiella pneumonia*e. Growth from urine was 349/985 (35 %) of which 310/349 (89 %) were *E. coli* while 39 (11 %) *K. pneumoniae.* High rates of antimicrobial resistance were detected among *E. coli* and Klebsiella isolates combined: sulphamethoxazole/trimethoprim 70 %, amoxicillin/clavulanate 36 %, chloramphenicol 20 %, ciprofloxacin 11 %, gentamicin 11 %, nitrofurantoin 4 %, ceftriaxone 3 %, piperacillin/tazobactam 27 %, cefoxitin 22 %, and cefepime 15 %. Multidrug resistance was noted in 33 % of the isolates. None of the isolates were resistant to imipenem. Overall, isolates from Kampala were more resistant to antimicrobials. Across the three districts combined, isolates producing beta-lactamase enzymes extended spectrum β-lactamase-(ESBL) and AmpC comprised 5.3 and 13.2 %, respectively. Further, medical procedures involving inoculation were independent risk factors [aOR 50.76 (1.80, 1432.90)] while residing in a rural district and use of sulphamethoxazole/trimethoprim 3 months prior to visiting the outpatient clinics were protective against carriage of multidrug resistant isolates. Furthermore, use of gentamicin was protective against AmpC producing isolates while clients attending HIV/AIDs clinics were less likely to carry such isolates. No factor was independently associated with carriage of ESBL-producing isolates.

**Conclusion:**

Antimicrobial resistance is prevalent among *E. coli* and *K.pneumoniae* carried in the gut of clients attending outpatient clinics in Kampala and two rural districts in Uganda. This could complicate treatment options for community-acquired infections caused by the *Enterobacteriaceae*.

**Electronic supplementary material:**

The online version of this article (doi:10.1186/s13104-016-2049-8) contains supplementary material, which is available to authorized users.

## Background

Antimicrobial resistance has become an issue of public health concern [1]; it is a major factor contributing to mortality and morbidity in settings with limited diagnostic facilities and treatment options. Fecal *Escherichia coli* is as an indicator of spread of acquired resistance-encoding genes in the community [2–4]. Klebsiella species on the other hand are opportunistic pathogens that play significant roles in hospital-acquired infections. Studies have shown that Klebsiella species have diverse resistance patterns to β-lactam agents. Although they are components of the normal flora, *E. coli* and Klebsiella species are considered potential causes of both community and hospital acquired infections [5].

Studies on commensal bacteria provide a more accurate reflection of the overall antimicrobial resistance burden in the population than data based on pathogenic isolates. In fact, studies on commensal bacteria in developing countries have shown high resistance rates to diverse antimicrobial agents [6]. In addition, drug resistance surveillance has revealed that asymptomatic carriers in the community are often colonized with resistant bacteria that subsequently lead to infection [7, 8]. Such studies are useful in the monitoring and understanding resistance mediated selection in populations [9].

In Uganda, antibiotics are readily available over the counter in community pharmacies [10], which portends significant rates of resistance among flora circulating within the community. In addition, inappropriate use of antimicrobials by pastoralists for animal diseases and the carriage of drug resistant potentially nosocomial bacteria in the livestock gut has been documented [11, 12]. Currently, most available data focuses on susceptibility of isolates from established infections without much attention on commensal isolates, particularly the Enterobacteriaceae [11, 12]. Furthermore, drug resistance is also prevalent in hospital settings in Uganda [13]. Moreover, the health care seeking behaviors in the community may vary depending on socioeconomic status of individuals involved [14]. Yet, information on resistance surveillance particularly for isolates from community settings is scarce as surveillance mainly focuses on susceptibility of isolates collected from clinical specimens in hospitals. The aim of this study was to characterize the antimicrobial susceptibility profiles of *E. coli* and Klebsiella species isolated as flora from faeces and urine of clients attending outpatient clinics in Uganda, and to investigate the factors associated with carriage of drug resistant isolates.

## Methods

### Study design

This was a cross-sectional study in which antimicrobial susceptibility testing was performed for *E. coli* and Klebsiella species isolated from stool or urine of asymptomatic clients attending outpatient clinics.

### Study sites and setting

Uganda comprises 111 administrative districts and one city, Kampala. Districts are further subdivided into counties and sub-counties. Uganda has three types of healthcare facilities; ‘Public’, ‘Private Not for Profit’ (PNFP) and ‘Private for Profit’. The Public and PNFP facilities are the most visited by Ugandans serving about 70 % of the population [15]. The levels of healthcare together with protocols guiding treatment are described elsewhere [10].

**Fig. 1:**
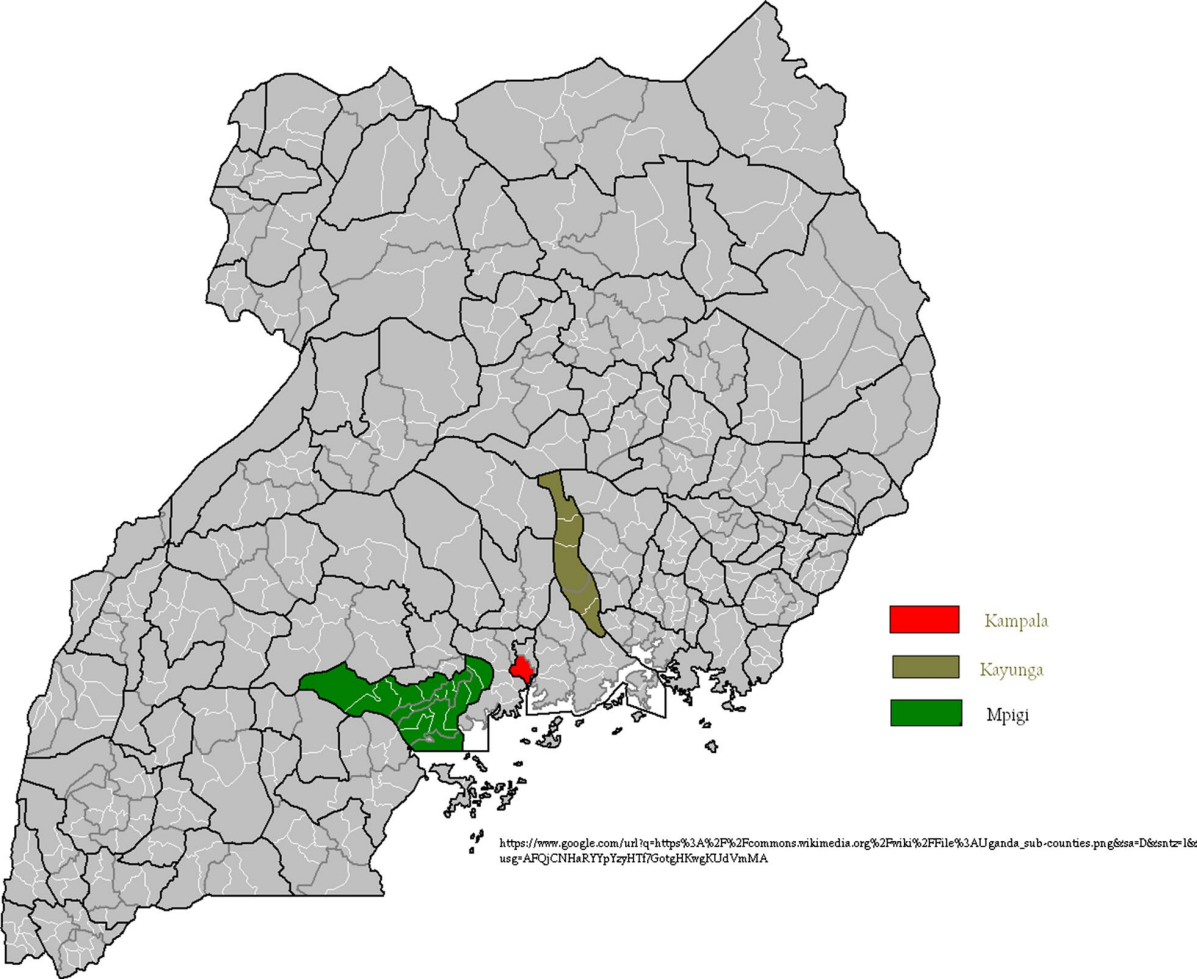
Study sites. The map was obtained from the URL to this figure is https://www.commons.wikimedia.org/wiki/File:Uganda_sub-counties.png, but slightly modified to emphasize the study sites

There were two study sites categorized ‘urban’ or ‘rural’ with varied distance and district human poverty index (HPI) [16]. The study districts were purposively selected with the assumption that urban areas favor increased exposure to antimicrobials and transmission of bacteria is less in areas of low humidity compared to those of high humidity [17]. The selected urban district and one of the rural districts are of wet tropical climate [18], while the other rural district is of wet and dry tropical climate. The urban site was Kampala district, which is the capital and largest city in Uganda with a HPI of 20.5. The rural sites included two districts in central Uganda namely, Kayunga 74 km and Mpigi 29.7 km from Kampala with HPI of 30.8 and 27.5, respectively, Fig. 1. The study was conducted among individuals attending outpatient clinics at 44 Public and PNFP hospitals or health centres including 16 in Kampala, 9 in Kayunga, and 19 in Mpigi.

### Study population and sample size estimation

The number of clients recruited from each facility was proportional to the contribution of the facility to the outpatient clinic attendance in the 3 months of April, May and June of the year 2006. The study was conducted in the second quarter of 2007 as a pilot in the urban district and the second quarter of 2008 for the two rural districts. All clients attending clinics were eligible to participate and the exclusion criteria was failure to provide either a stool or urine sample.

In Uganda no data exists on antimicrobial resistance among *E. coli* and/or *Klebsiella pneumoniae* in well-defined community infections. As such, the sample size was computed based on the prevalence of *K. pneumoniae* carriage in urine which has been observed to be about 19.6 % among clinical samples at clinical microbiology laboratory, College of Health Sciences, Makerere University (unpublished observations). The formula N = [(Z_(α/2)_)^2^ *P *(1 − p)]/d^2^ = 242 where; Z = 1.96 (the standard normal deviation at 95 % CI), P = estimated prevalence of the problem under study (P = 0.196), d = margin of error (precision) of prevalence estimate at 5 % (0.05), α = level of significance at 5 % and n = required sample size was used. Primary sampling clusters were used to recruit individuals and thus, using a design effect of 2.0, the sample size under cluster sampling was 484 per district and 1452 for the three districts combined.

### Selection of health care facilities and participants

**Fig. 2:**
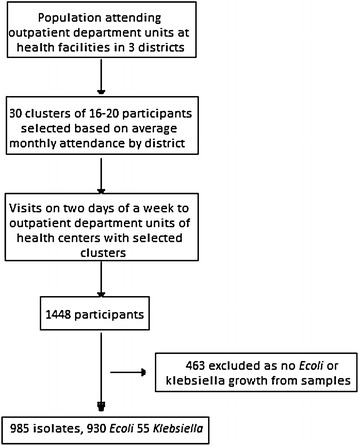
Study flow chart

In each of the three districts multistage sampling was done based on the average clinic attendance for each district to ensure a sample size of 484, given an observed frequency of Klebsiella species in 19.6 % of urine specimens and a design effect of 2, Fig. 2. Thirty clusters of 16–20 participants were selected from each district using the probability proportion to size sampling, which ensured all individuals in the target population had an equal chance of being selected. Two busy days of a week were purposively selected to visit each health care facility. When the number exceeded 20, systematic sampling was carried out.

### Data collection

A standardized interviewer administered questionnaire (Additional file 1: Figure S1) was used to collect data on socio-demographics (age, sex, and residence), heath care unit, and reason for visit and history of use of antimicrobial agents, history of admission and history of medical procedures in the previous 3 months.

#### Sample collection

Participants were instructed to provide a stool or a urine sample if they were unable to provide stool. The specimens were collected in sterile containers with screw caps. Samples from rural districts were stored at 4 °C for one to 3 days prior to transportation by road in cool boxes while those from Kampala were transported to the laboratory on the day of collection.

### Cultures

Isolation of *E. coli* and Klebsiella species and the antimicrobial susceptibility testing were performed at the Clinical Microbiology Laboratory, College of Health Sciences, Makerere University. The laboratory participates in the College of American Pathologist bacteriology external quality assurance scheme (CAP no. 7225593).

Samples were streaked onto MacConkey agar and incubated for 18–24 h at 37 °C in ambient air. In case of stool, specimens were first emulsified in sterile normal saline before inoculation of the MacConkey plates. Lactose fermenting isolates, from the fourth or third streak area with colonial morphologies suggestive of *E. coli* and Klebsiella species were subjected to an oxidase test, and when negative, they were cultured for 18–24 h on the following media: triple sugar and iron (TSI) agar, Simmon’s citrate agar, and sulphide-indole-motility (SIM) media for identification. Following identification, *E. coli* and Klebsiella isolates with inconclusive identification were confirmed with the API 20E system (BioMerieux Marcy 1’Etoile, France).

### Antimicrobial susceptibility testing

Drug susceptibility testing (DST) was performed with the disc diffusion method on Mueller Hinton Agar (MHA) (Biolab, Hungary) plates as recommended by the clinical laboratory standards institute (CSLI). Three colonies were emulsified into sterile saline and the turbidity of the suspension adjusted to the 0.5 McFarland standard. The antimicrobial disks (Biolab, Hungary) used included ampicillin (10 µg), amoxicillin/clavulanate (20/10 µg), cefuroxime (30 µg), ceftriaxone (30 µg), cefotaxime (30 µg), ceftazidime (30 µg), meropenem (30 µg), trimethoprim/sulfamethoxazole (1.25/23.75 µg), chloramphenicol (30 µg), gentamicin (10 µg), ciprofloxacin (5 µg), nitrofurantoin (300 µg), cefepime (30 µg), piperacillin/tazobactam (100/10 µg) and cefoxitin (30 µg).

*Escherichia coli* ATCC 25992/ATCC 35218, *Staphylococcus aureus* ATCC 25923, and *Pseudomonas aeruginosa* ATCC 27853 were used to quality control the susceptibility testing procedures in line with CLSI guidelines.

For isolates resistant to second or third generation cephalosporins, minimum inhibitory concentrations (MICs) were performed by E-test (AB BIODISK Solna, Sweden) according to the manufacturer’s instructions and these were confirmed using the Vitek 2 identification and susceptibility system (Biometric, Marcy I’Etoile, France). The results were interpreted according to CLSI guidelines [19].

### Detection of extended spectrum β-lactamase production

Detection of extended spectrum β-lactamases (ESBLs) was performed using the double disc synergy test (DDST) using amoxicillin/clavulanate, ceftazidime, ceftriaxone, aztreonam, and cefotaxime as previously described [20, 21]. E-test strips (containing cefotaxime/ceftotaxime-clavulanate, ceftazidime/ceftazidime-clavulanate and cefepime/cefepime-clavulanate) (AB BIODISC, Solna, Sweden) were used. *K. pneumoniae* 70,063 was used to control ESBL detection. The E test ESBL strip (AB Biodisk, Solna, Sweden) carries two gradients: on one end, ceftazidime or cefotaxime; and on the opposite end, ceftazidime or cefotaxime plus clavulanic acid. MIC were interpreted as the point of intersection of the inhibition ellipse with the E test strip edge. A ratio of ceftazidime or cefotaxime MIC to ceftazidime or cefotaxime-clavulanic acid MIC equal to or greater than eight indicated the presence of ESBL (manufacturer’s instructions) [22].

### Phenotypic detection of AmpC enzymes

Phenotypic detection of AmpC β-lactamase production was performed on isolates with reduced cefoxitin diameters (≤17 mm). These were sub-cultured on MHA containing 8 μg/ml of cefoxitin agar (USP reference standard, Rockville) and screened with E-test strips (cefotetan/cefotetan-cloxacillin, CN/CNI AB BIODISC, Solna, Sweden). A ratio of ≥8, deformation of the ellipse, or the presence of a phantom zone were interpreted as positive for AmpC production [23].

### Data analysis

Questionnaire responses and DST data were checked for completeness. Data was double entered for validation using EPIDATA software, cleaned and exported to STATA (v10) for analysis. Data were analyzed using descriptive statistics, frequencies and bivariate analyses (cross-tabulations). Associations were tested using Pearson’s Chi square. A significant level was set at p = 0.05. Odds ratios (OR) were determined between socio-demographic variables, health facility level, district, reason for visit, prior use of antimicrobial, history of hospital admission and medical procedures 3 months prior to visits, with the following outcomes; (1) resistance to one antibiotic, (2) resistance to three different classes of antimicrobial agents -multi-drug resistance (MDR), (3) ESBL phenotype, (4) AmpC phenotype.

Variables with p < 0.15 were entered into multivariate logistic regression models with backward elimination. To control for the effect of clustering, regression with robust standard errors was used. Independent variables used were; sex (male versus female), health center level, health sub-district and district, history of admission, procedures and antibiotic use recalled by client and from health record, in the previous 3 months.

Differences between proportions of resistant isolates were determined to compare variation within districts using MDR as surrogate marker. MDR was determined based on categories and drugs used to determine susceptibility among the *Enterobacteriaceae* [24]. The categories included: aminoglycosides (gentamicin); anti-pseudomonal penicillin/β-lactamase inhibitor (piperacillin/tazobactam); carbapenem (meropenem); non-extended spectrum cephalosporins (cefuroxime); extended spectrum cephalosporin (ceftriaxone, ceftazidime, cefotaxime, cefepime); cephamycins (cefoxitin); fluoroquinolone (ciprofloxacin); folate pathway inhibitors (sulphamethoxazole/trimethoprim); penicillins (ampicillin); and phenicols (chloramphenicol). These 11 antimicrobial agents represented 11 of the 16 recommended categories however, second and third generation cephalosporins were considered as oxyminocephalosporins.

## Results

A total of 1448 participants were recruited; 474 (33 %) Kampala, 508 (35 %) Kayunga, and 466 (32 %) Mpigi. Females were 913 (63.3 %) while men were 529 (36.7 %) and the difference was statistically significant (p = 0.005). The mean age of the participants was 24.8 years (SD 17.5) (Table 1); 802 (55.7 %) of them belonged to the 15–44 year age group. Participants provided 730 and 718 stool and urine samples, respectively, which were cultured for identification of *E. coli* and Klebsiella isolates.

**Table 1:** Characteristics of participants attending OPDs in the three study sites

	Kampala n (%)	Kayunga n (%)	Mpigi n (%)	Total n (%)	p value
Sex					
Female	324 (68.8)	297 (58.8)	292 (62.7)	913 (63.3)	
Male	147 (31.2)	208 (41.2)	174 (37.3)	529 (36.7)	0.005
Age—years (SD)	23.5 (15.4)	22.5 (16.5)	28.8 (19.7)	24.8 (17.5)	
Age (years)—groups					
0–14	121 (25.7)	196 (38.9)	126 (27.0)	443(30.7)	
15–44	310 (65.8)	249 (49.4)	243 (52.2)	802 (55.7)	
45+	40 (8.5)	59 (11.7)	97 (20.8)	196 (13.6)	<0.001
Health centre level					
National referral hospital	197 (41.6)	0 (0.0)	0 (0.0)	197 (13.6)	
General hospital	152 (32.1)	114 (22.4)	158 (33.9)	424 (29.3)	
HCIV	17 (3.6)	150 (29.5)	50 (10.7)	217 (15.0)	
HCIII	108 (22.8)	244 (48.0)	258 (55.4)	610 (42.1)	<0.001
Specimen					
Stool	167 (35.2)	469 (92.3)	94 (20.2)	730 (50.4)	
Urine	307 (64.8)	39 (7.7)	372 (79.8)	718 (49.6)	<0.001
Prescription					
Yes	319 (67.3)	383 (75.4)	391 (84.1)	1093 (75.5)	
No	155 (32.7)	125 (24.6)	74 (15.9)	354 (24.5)	<0.001
Reason for visit					
ISS	24 (5.1)	55 (11.7)	43 (9.6)	122 (8.8)	
Infection	268 (57.3)	227 (48.2)	236 (52.6)	731 (52.7)	
General	176 (37.6)	189 (40.1)	170 (37.9)	535 (38.5)	0.003
History of admission					
Yes	41 (8.7)	9 (1.8)	75 (16.5)	125 (8.7)	
No	433 (91.4)	494 (98.2)	379 (83.5)	1306 (91.3)	<0.001
History of medical procedures					
Contact	17 (30.4)	0 (0.0)	0 (0.0)	17 (13.1)	
Inoculation	34 (60.7)	17 (89.5)	37 (67.3)	88 (67.7)	
Surgery	5 (8.9)	2 (10.5)	18 (32.7)	25 (19.2)	<0.001

*HCIII* health center III, *HCIV* health center IV, *ISS* immunosuppressive syndrome

### Socio-demographic factors of participants

Five hundred and thirty-five participants visited the clinics for general conditions and these comprised headache and fever (422, 28.49 %); follow-up review (85, 5.74 %); antenatal care 20 (1.35 %); and cardiovascular diseases (15, 1.01 %). Other reasons for visits included infections (731, 52.7 %), such as respiratory tract infections (312, 21.07 %); gastro-intestinal (309, 20.86 %); genital urinary infections (125, 8.44 %); skin infections (64, 4.32 %); and attending HIV/AIDS clinics (122, 8.71 %). Use of antimicrobials 3 months prior to the study was high (1093, 75.5 %); septrin (774, 70.6 %); penicillins (369, 33.9 %), ciprofloxacin (130, 11.9 %); and gentamicin (45, 4.1 %), Table 1. One hundred and twenty-five (125, 8.7 %) participants had been admitted to hospital 3 months prior to the clinic visit while 130 (8.9 %) underwent medical procedures, of which 17 (13.1 %) involved only contact (such as radiological investigations) while 88 (67.7 %) had investigations like taking blood for malaria, injections and infusion (inoculation), and 25 (19.2 %) had minor or major surgery. Overall, there was significant variation among the parameters related with resistance across the three districts, Table 1.

### Prevalence of *E. coli* and Klebsiella species

Of the 1448 participants, growth of *E. coli* or *K. pneumoniae* was obtained from 985 samples and significant variation was observed across the three districts; 274 Kampala, 406 Kayunga, and 305 Mpigi (p < 0.001). Growth from stool occurred in 636 (87 %) samples of which 620 (97 %) were *E. coli* and 16 (3 %) were *K. pneumonia*e. Growth from urine occurred in 349 (49 %) samples of which 310 (89 %) were *E. coli* and 39 (11 %) *K. pneumoniae;* none of the urine samples had growth of ≥10^4^ colony forming units (CFU) implying there was no infection-related growth. Forty-one samples grew both *E. coli* and *K.pneumoniae*; where both grew, *E. coli* was considered as it is the commonest cause of community infections. Overall 930 (94.4 %) *E. coli* and 55 (5.6 %) *K. pneumoniae* isolates were isolated and studied.

### Susceptibility profiles

Overall, two hundred and seven (207, 21 %) isolates were susceptible to all antimicrobial agents tested;

1. β-lactam agents; 617 (67 %) *E. coli* isolates were resistant to ampicillin (Klebsiella were omitted as they are inherently resistant to ampicillin). Resistance to other drugs was (for both *Klebsiella* and *E. coli*); 145 (14 %) cefuroxime, 29 (3 %) ceftriaxone, and 359 (36 %) amoxicillin/clavulanate. Further analysis revealed resistance in 263 (27 %) isolates for piperacillin/tazobactam, 213 (22 %) cefoxitin and 152 (15 %) cefepime, Table 2. Although none of the isolates was resistant to meropenem, zone diameters of ≤ 25 mm were noted in 167 (17 %) of the isolates a finding that necessitates screening for carbapenamase activity.

**Table 2:** Antimicrobial resistance profiles of *E. coli* and *Klebsiella pneumoniae* in the three districts

Antimicrobial	Districts				p value (variation across the districts)
	Kampala n/N (%)	Kayunga n/N (%)	Mpigi n/N (%)	Total n/N (%)	
Ampicillin^a^	224/258 (86.8)	218/396 (55.1)	175/275 (63.9)	617/928 (66.5)	<0.001
Amoxicillin-clavulanate	184/274 (67.2)	80/406 (19.7)	88/304 (29.0)	352/984 (35.8)	<0.001
Cefuroxime	43/273 (15.8)	77/405 (19.0)	22/304 (7.2)	142/982 (14.5)	<0.001
Ceftriaxone	16/274 (5.8)	2/406 (0.5)	10/303 (3.3)	28/983 (2.9)	<0.001
Cefotaxime	14/273 (5.1)	2/406 (0.5)	6/302 (2.0)	22/981 (2.2)	<0.001
Ceftazidime	15/273 (5.5)	2/406 (0.5)	8/304 (2.6)	25/983 (2.5)	<0.001
Cefepime^b^	93/178 (52.3)	26/76 (34.2)	29/91 (31.9)	148/345 (42.9)	0.001
Ciprofloxacin	73/274 (26.6)	15/405 (3.7)	17/304 (5.6)	105/983 (10.7)	<0.001
Sulfamethoxazole-trimethoprim	234/272 (86.0)	262/405 (64.7)	189/304 (62.2)	685/981 (69.8)	<0.001
Gentamicin	68/273 (24.9)	19/406 (4.7)	18/303 (5.9)	105/982 (10.7)	<0.001
Nitrofurantoin	23/270 (8.5)	4/403 (1.0)	8/304 (2.6)	35/977 (3.6)	<0.001
Chloramphenicol	83/273 (30.4)	54/405 (13.3)	56/301 (18.6)	193/979 (19.7)	<0.001
Cefoxitin^b^	106/181 (58.6)	40/78 (51.3)	63/88 (71.6)	209/(60.2)	0.023
Piperacillin-tazobactum^b^	126/173 (72.8)	64/79 (81.0)	67/93 (72.0)	257/345 (74.5)	0.315
Meropenem	0/273 (0.0)	0/403 (0.0)	0/300 (0.0)	0/976 (0.0)	–

^a^Only *Escherichia coli*

^b^Only amoxicillin clavulanic acid resistant isolates tested
2. Other antimicrobial agents. High resistance against sulphamethoxazole/trimethoprim (698, 69 %), chloramphenicol (19 %, 195), ciprofloxacin (107, 11 %), gentamicin (106, 11 %), and nitrofurantoin (40, 4 %) was detected. Overall, resistance did not vary by gender except that isolates from females 123/167 (74 %) were more likely to have reduced susceptibility (inhibition zone diameter ≤25) to carbapenems (p < 0.023).
3. MDR was noted in 356 (36.1 %) isolates and there was significant variation across districts (p < 0.001). Three hundred and twenty-three *E. coli* isolates (323/930, 34.7 %) and 33 (33/55, 60 %) Klebsiella isolates were MDR and the finding was statistically significant (p < 0.001). The highest MDR prevalence was observed in Kampala, an urban district. MDR phenotype also varied by health sub-districts (HSD) ranging from 9/46 (19.6 %) in rural to 26/33 (79 %) in urban. Further, the MDR phenotype was detected in all health care facilities in Kayunga (9) and Kampala (16), and was not detected in 4 of the 19 health care facilities in Mpigi. Among the Ampicillin resistant isolates, 32 (3 %) were resistant to nitrofurantoin, 94 (9 %) gentamicin, 100 (10 %) ciprofloxacin, 192 (19 %) chloramphenicol and 592 (60 %) septrin. In addition, there was high resistance among the isolates to multiple drug combinations, Table 3.

**Table 3:** Frequency and patterns of resistance phenotypes among isolates from the study sites

Number of drug classes	Drug combination	Number of isolates resistant to drug combination
3	Ampicillin + sulphamethoxazole-trimethoprim + chloramphenicol	183
	Ampicillin + sulphamethoxazole-trimethoprim + ciprofloxacin	92
	Ampicillin + sulphamethoxazole-trimethoprim + gentamicin	88
	Ampicillin + sulphamethoxazole-trimethoprim + nitrofurantoin	22
4	Ampicillin + sulphamethoxazole-trimethoprim + quinolone + aminoglycoside	57
5	Ampicillin + sulphamethoxazole-trimethoprim + quinolone + aminoglycoside + nitrofurantoin	9
6	Ampicillin + sulphamethoxazole-trimethoprim + quinolone + aminoglycoside + nitrofurantoin + chloramphenicol	6
7	Ampicillin + sulphamethoxazole-trimethoprim + quinolone + Aminoglycoside + nitrofurantoin + chloramphenicol + oxyminocephalosporins	5

### Prevalence of ESBL-producing isolates

Overall, 209 bacterial isolates from 40 health care centers qualified for AmpC screening (based on cefoxitin resistance) while 63 isolates from 23 health centers qualified for ESBL-screening (based on CLSI guidelines). AmpC and ESBL-producing isolates were higher in Kampala [ESBL; 35/274 (12.8 %), AmpC; 65/274 (23.7 %)] compared to Kayunga [ESBL 7/406 (1.7 %), AmpC; 27/406 (6.7 %)] and Mpigi [10/305 (3.3 %), AmpC; 37/305 (12.1 %)]. However, among isolates that qualified for screening for ESBLs, there was no significant difference in the distribution of the enzymes across the three districts i.e. for ESBLs in 35 (83.3 %), 7 (77.8 %) and 10 (83.3 %) isolates (p = 0.886) and AmpC in 65 (53.3 %), 27 (46.6 %) and 37 (55.2 %) isolates (p = 0.505); for both enzymes in Kampala, Kayunga and Mpigi, respectively.

AmpC enzymes were detected in 61 % (116/191) and 72 % (13/18) *E. coli* and Klebsiella, respectively (χ^2^ (1) 4.9527, p = 0.026). ESBLs were detected in 81 % (42/52) *E. coli* and 100 % (10/10) of the screened Klebsiella isolates. The coexistence of ESBLs and AmpC enzymes was detected in 28.8 % (15/52) of the isolates. The cefoxitin inhibition zone diameters of ≤13 mm were significantly associated with presence of AmpC enzymes in both *E. coli* and Klebsiella isolates (p < 0.038) but it was not the case for ESBLs when reduced zone diameters recommended for ESBL screen (CLSI) were used (p = 1.00).

The overall prevalence of AmpC phenotype was 13 % (129/985) for the three districts. Whereas 97 of the 182 MDR screened had AmpC enzyme, this was not statistically significant [χ^2^ (1) = 0.0253, p = 0.874]. Isolates bearing AmpC β-lactamase were sensitive to gentamicin 96/129 (74 %) [χ^2^ (1) 4.766, p < 0.029] while 74 % (90/122) of them were resistant to piperacillin/tazobactam much as this association was not significant [χ^2^ (1) 0.766, p = 0.381].

The prevalence of the ESBL phenotype was 5.3 % (52/985). Among isolates bearing ESBLs β-lactamase and were resistant to amoxicillin/clavulanate, 86 % (32/37) were also resistant to piperacillin/tazobactam [χ^2^ (1) 0.087, p = 0.767].

### Univariate analysis and logistic regression model

On univariate analysis, significant risk factors for carriage of ESBLs among outpatient clinic attendees were Health center level (p = 0.037) and use of antibacterial agent in the last 3 months prior to clinic visit (p = 0.023), Additional file 2: Table S1. In addition to these the health sub-districts with less than 100 participants with growth (p = 0.079) and use of septrin (p = 0.088) were the variables included in the logistic regression model. Using backward stepwise procedure, no independent risk factor for carriage of ESBL enzymes was identified. However, without controlling (for other factors), use of antimicrobials 3 months prior to clinic visit was found to be associated with carriage of ESBL cOR 4.47 (95 % CI 1.15, 17.44), Table 4.

**Table 4:** Unadjusted and adjusted Odds ratios (OR) of ESBLs, AmpC and MDR by participants characteristics

Characteristics	ESBL		AmpC		MDR	
	cOR (95 % CI)	aOR (95 % CI)	cOR (95 % CI)	aOR (95 % CI)	cOR (95 % CI)	aOR (95 % CI)
Age						
0–14					1.0	1.0
15–44					1.65 (1.22, 2.23)^a^	6.49 (0.91, 46.16)
45+					1.38 (0.90, 2.12)	1.89 (0.21, 16.75)
Sex						
Female			1.0	1.0		
Male			1.52 (0.88, 2.63)	1.51 (0.76, 2.97)		
HC level						
NR	1.0	1.0			1.0	1.0
GH	1.65 (0.35, 7.8)	0.89 (0.15, 5.28)			0.32 (0.20, 0.52)^a^	0.18 (0.001, 27.32)
HCIV	0.53 (0.07, 3.98)	0.31 (0.03, 2.88)			0.27 (0.16, 0.46)^a^	1.96 (0.01, 491.00)
HCIII	–	–			0.21 (0.13, 0.33)^a^	2.59 (0.02, 331.66)
District						
Kampala					1.0	1.0
Kayunga					0.17 (0.12, 0.24)^a^	0.02 (0.00, 3.54)
Mpigi					0.20 (0.14, 0.28)^a^	0.36 (0.003, 38.44)
HSD						
Cases >100	1.0	1.0			1.0	1.0
Cases <100	6.77 (0.81, 56.94)	6.71 (0.46, 97.96)			1.75 (1.33, 2.29)^a^	0.13 (0.001, 12.37)
Reason for visit						
ISS			1.0	1.0		
Infection			1.38 (0.44, 4.35)	1.72 (0.47, 6.35)		
General			3.42 (1.05, 11.12)^a^	4.38 (1.14, 16.84)^a^		
History of admission						
Yes			2.76 (1.12, 6.84)^a^	2.92 (0.95, 9.02)		
History of medical procedures						
Contact					1.0	1.0
Inoculation					4.11 (0.45, 37.69)	50.76 (1.80, 1432.98)^a^
Surgery					0.83 (0.06, 11.42)	2.48 (0.06, 99.30)
Antibiotic use						
Yes	4.47 (1.15, 17.44)^a^	4.57 (0.90, 23.20)			0.79 (0.58, 1.07)^a^	
Use of gentamicin						
Yes			0.27 (0.05, 1.32)	0.17 (0.03, 0.95)^a^		
Use of ciprofloxacin						
Yes					1.52 (0.96, 2.40)^a^	5.71 (0.69, 47.01)
Use of septrin						
Yes	4.33 (0.84, 22.47)	4.70 (0.33, 66.82)			0.53 (0.40, 0.71)^a^	1.92 (0.45, 8.23)

The analysis for association of predictor and outcome (p ≤ 0.15)

*NR* national referral, *GH* General hospital, *HCIV* health center 4, *HCIII* health center III, *HSD* health sub-district, *cOR* crude odds ratio, *aOR* adjusted odds ratio, *ISS* immune suppression syndrome (HIV/AIDS)

^a^Significant association

Significant risk factors at univariate analysis for carriage of AmpC enzymes were reason for coming to clinic (p = 0.003), history of admission (p = 0.023) and history of medical procedures (p = 0.044), Additional file 2: Table S1. These variables, together with sex (p = 0.132) and use of gentamicin (p = 0.100) were included in the model. Following backward stepwise procedure, visiting the outpatient clinic for reasons other than infection or HIV/AIDs care [aOR 3.56 (95 % CI 1.08, 11.77)] was an independent risk factor for carriage of AmpC producing *E. coli* and *K.pneumoniae* while use of gentamicin was independent of such carriage [aOR 0.17 (95 % CI 0.03–0.95]. However, without controlling for other factors, history of admission was found to be associated with carriage of AmpC [cOR 2.76 (95 % CI 1.12, 6.84)], Table 4.

MDR isolates were prevalent across the three districts and in the univariate analysis the significant factors for carriage of MDR isolates were age group (p = 0.004), health center level (p < 0.001), health sub-district (p < 0.001), district of residence (p < 0.001), health sub-district (group of <100 participants with growth (p < 0.001), Additional file 2: Table S1, and use of septrin (p < 0.001), Additional file 2: Table S1 (Additional file 3: Table S2). In multivariate analysis four variables; age group, health center level, district of residence, and use of septrin together with history of medical procedures (p = 0.075), use of antimicrobial agents (p = 0.122); including use of ciprofloxacin (p = 0.071) and were included in the model. Using backward stepwise procedure, having had medical procedures involving inoculation in the last 3 months prior to visiting health facility was independent risk factor for carriage of MDR isolates [aOR 50.76 (95 % CI 1.80–1432.90)]. However, without controlling for other factors when compared to residence in Kampala, residing in Kayunga and Mpigi was protective by 83 % (95 % CI 0.11–0.26) and 80 % (95 % CI 0.13, 0.32), respectively and septrin use was protective by 47 % (95 % CI 0.40, 0.71) of carriage of MDR *E. coli* or *K. pneumoniae*. Furthermore, the age group 15–44 was 1.65 times more likely to have MDR isolates (95 % CI 1.22, 2.23) compared to participants less than 15 years and HSD with less than 100 cases were 1.75 times more likely to have such isolates (0.5 % CI 1.33–2.29). Also, attending lower hospitals was protective from carriage of MDR isolates when compared to National referral hospitals [cOR 0.32 (95 % CI 0.20, 0.52)] for general hospitals, HCIV [cOR 0.27 (95 % CI 0.16, 0.46)] and HCIII [cOR 0.21 (95 % CI 0.13, 0.33)], Table 4.

## Discussion

We have reported the prevalence and susceptibility patterns of *E. coli* and *K. pneumoniae* isolated from the intestinal and urinary tracts of individuals attending urban and rural outpatient clinics in Uganda. In order to study susceptibility of flora among outpatient department (OPD) clients for the first time, the study was conducted in two phases a pilot study that took place between May and June of 2007, and involved generating sampling frame, seeking permission and recruitment took place in Kampala district. The aim was to test the recruitment strategy and sample collection, transportation and analysis. The experience from the pilot study was then used for study activities in the two rural districts.

The findings in this study reveal high rates of resistance to commonly used antibiotics such as ampicillin and septrin and relatively lower resistance rates to amoxicillin/clavulanate, chloramphenicol, ciprofloxacin, gentamicin, nitrofurantoin and ceftriaxone. These findings are similar to those from neighboring Kenya [25]. The high levels of resistance to piperacillin/tazobactam and cefoxitin among the amoxicillin/clavulanate resistant isolates found were associated with AmpC enzymes. Despite the low resistance to third generation cephalosporins, ESBLs were detected in 5.3 % isolates most of which were MDR with co-resistance to ciprofloxacin, septrin and gentamicin. However, the MDR rates in our study was lower than that of fecal carriage among outpatient department volunteers in Cameroon [26]. This study also found that *E. coli* is the species most commonly isolated among ESBL producers [26]. The prevalence of ESBLs in our study was similar to findings among French Guiana Amerindians living in a very remote village [27]. There was significant variability among the three districts, one possible reason could be the nature of samples provided, when individuals visit outpatient clinics, the convenient specimen depends on the respective toilet habits of participants and possibly diet, however the nature of bathrooms at the clinic may not be conducive for a participant to take much time there in. In Uganda communities with the wet and dry climate tend to have diets different from those of wet climate. The variation in sex distribution cannot be explained, neither is the presence of more elderly people in the rural district of Mpigi, this same district prescribed more ciprofloxacin, and gentamicin, had higher admissions and surgeries too.

### Susceptibility patterns

The resistance to antibacterial agents tested significantly varied across the three districts and was highest in the urban district of Kampala, save for cefoxitin where it was higher in the western rural district of Mpigi. Resistance to piperacillin/tazobactam did not significantly vary across the districts but was highest in the eastern rural district of Kayunga. Our findings are consistent with previous studies that reported higher resistance in urban compared to rural areas [28, 29]. Resistance also varied within districts possibly reflecting differences in services offered by health care givers. It is also possible that segregation in community activities where some are entirely agricultural based with sparse population lessens access to antibiotics. Populations having commercial activity and higher population density create opportunity for increased environmental density of antibiotic use and antibiotic resistant bacteria as a result of access to drug outlets and health facilities [28].

In this study, over 76 % of participants reported prior use of antimicrobial agents particularly septrin and penicillins. The prevalence of HIV in Uganda necessitates use of antimicrobials for treatment of opportunistic infections, and the routine use of septrin prophylaxis against *Pneumocystis jirovecii* pneumonia. This could contributes to bacterial resistance [30, 31]. However in this study the use of septrin was protective against carriage MDR *E. coli* and *K. pneumoniae*. This finding requires focused studies to determine factors underlying this protection. Furthermore, individuals with HIV/AIDs were significantly less likely to have *E. coli* and *K. pneumoniae* producing AmpC enzymes; this may be attributed to the decrease in infections attributed to use of basic care package (BCP), a patient managed, home based care system introduced since 2005 and is widely used [32]. It empowers HIV-positive people to avert opportunistic infections and exposure to malaria transmitting mosquito among many other benefits; thus living a healthier life and preventing occurrence of diseases [33, 34]. This possibly keeps these individuals away from frequent exposure to health facilities, a factor likely to be contributing to carriage of AmpC in this study, and visiting drug outlets thus indirectly protecting them from exposure to AmpC-bearing *Enterobacteriaceae*. There was little use of third generation cephalosporins, β-lactamase inhibitors and cephamycins and yet resistance to the respective classes was detected among study participants. There is a possibility that use of antimicrobial agents to which bacteria are already resistant selects for further resistance and also co-selects resistant determinants that may be co linked to resistance of the respective drug in use.

The resistance to cefepime was noted to be higher than that of ceftriaxone, such resistance may be due to presence of oxacillinase enzymes like Oxa-30 and Oxa-31, one of the derivatives of Oxa-1, that confers a spectrum sparing ceftazidime and cefotaxime and hydrolyses cefepime, but it has also been reported to be associated with alteration or loss of outer membrane porins [35, 36]; these possibilities should be explored in subsequent studies.

The commonest MDR combination when associated with beta-lactamase enzymes determinants are likely to be selected for by use of respective antimicrobials, thus increasing the magnitude of beta-lactamases as postulated elsewhere [37]. The health centers studied were assumed to serve over 70 % of the population. However there is inadequate laboratory diagnosis in most of the rural district areas often leaving resistance to go undetected. Under these circumstances, empiric as opposed to targeted therapy prevails [38]. In the period of the study, visits to health facilities depended on availability of medicines and stock outs were frequent consistent with findings from previous studies [39]. When the antimicrobials were not present, clients may have resorted to drug outlets [40] where doses offered are suboptimal [10] and such exposure is an established driver of resistance. Frequent use of antimicrobial creates a pool of resistant commensal bacteria that contribute to general increase and dissemination of bacterial resistance worldwide [41]. In this setting, this is compounded by the liberal use of antimicrobials by farmers [12]. Evidence from settings where antibacterial agents are accessed by prescription only, have shown that individuals prescribed an antibiotic in primary care develop bacterial resistance to the respective antibiotic, with effect being greatest in the month immediately after treatment but persistence may go on for over 12 months [42]. The high presence of septrin resistance in a setting where it is continuously used, may be explained by several of the above factors.

### Factors associated with drug resistance

Multidrug resistance was common and factors such as age, level of health facility, location of health sub-district, district of residence, undergoing medical procedures and use of septrin were associated with multidrug resistance. However, only undergoing medical procedures associated with inoculation was an identified risk factor for carriage. Other factors yet to be identified contribute to the use of septrin within 3 months prior to visiting the clinics [cOR 0.53 (95 % CI 0.40, 0.71)] and district of residence whereby those from rural districts of Kayunga [cOR 0.17 (95 % CI 0.11, 0.26)] and Mpigi [cOR 0.20 (95 % CI 0.13, 0.32)] were protected from carriage of MDR flora compared to those who reside in Kampala. Furthermore visiting lower health centers like general hospital [cOR 0.32 (95 % CI 0.20, 0.52)], HCIV [cOR 0.27 (95 % CI 0.16, 0.46] and HCIII [cOR 0.21 (95 % CI 0.13, 0.33)] compared to national referral hospital was protective of MDR carriage, the age group 15–44 was a risk factor for carriage of MDRisolates compared to participants ≤15 years [cOR 1.65 (95 % CI 1.22, 2.23)] and so were HSD of less than 100 cases cOR 1.75 (95 % CI 1.33, 2.29).

Multidrug resistance was noted for over 60 % of ESBLs and 70 % AmpC bearing isolates; whereas no factor was independently associated with carriage of isolates producing ESBLs, having used antibacterial agents during 3 months prior to OPD visit may have combined with yet unknown factors to contribute to carriage of flora with the enzyme [cOR 4.47 (95 % CI 1.15, 17.44)]. Previously studies have reported that antibiotic exposure affects ESBL faecal carriage in the community [27].

The isolates with AmpC enzymes were independently associated with clinic visits for reasons other than infection or HIV/AIDs care [aOR 4.38 (95 % CI 1.14, 16.84)] while use of gentamicin was protective of such carriage [aOR 0.17 (95 % CI 0.03, 0.95)]. Participants with history of admission 3 months prior to admission were also likely to have carriage of isolates with AmpC enzymes [cOR = 2.76 (95 % CI 1.12, 6.84)]. Such findings highlight the role played by direct interaction of clients with facility environment including health care givers. The association of hospital admission and care with AmpC and MDR isolates can be explained by the levels of overcrowding in health facilities compounded by substandard infection control practice in the study setting where hand hygiene is seen more as self-protection by health care givers as opposed to breaking the chain of transmission in the facility [43]. Such practice facilitate transmission of bacteria between clients and care givers [44]. This argument is strengthened by finding procedures associated with inoculation, where direct contact with health care providers is inevitable, as an independent risk factor.

Previous studies have pointed out the acquisition of AmpC enzyme bearing bacteria from health facilities. However the presence of these enzymes in the community following the introduction by those who visited health facilities is worrying [45] as they drive the use of Carbapenems for community-acquired infections [46] and are associated with poor outcome [46].The presence of MDR isolates in varied combination some covering over five different class drugs may be an indicator of evolving gene pools in flora from the study areas [47]. In this study age and sex were not independently associated with resistance.

Population exposure to antibiotics is related to ESBL community carriage rates [27]. However several studies in the past [28] and a recent one from Australia demonstrated presence of resistance among individuals who have not taken antibiotics [48]. The presence of ESBL enzymes and associated MDR among individuals reporting to health centers without associated factors has been highlighted in other studies [49], and several sources for ESBL transmission in the community have been identified. For example ESBLs was detected from food of animal origin such as raw meat similar to faecal samples from hospitals in The Netherlands [50]. Extended-spectrum β-lactamase genes of *E. coli* was detected in chicken meat and humans, in the Netherlands [50]. Similarly in Uganda the resistance of *E. coli* isolates from faecal samples of chicken and pigs support the possibility of having resistance arising from the food chain [11]. Transmission of bacteria between human and animal sources has been reported from communities associating with wild life in Uganda [51] and in Portugal Enterobacteriaceae from health humans had resistance determinants similar to those from food producing animals [52]. Elsewhere in Kenya companion animals of Pastoralists were found to carry ESBls [53] and there is a possibility that similar scenarios of transmission of resistance take place in this setting.

The environment has been suggested to serve as a reservoir for ESBL genes since isolates from varied sources are noted to be similar to those of humans [54]. A variety of factors such as community hygiene levels, people’s behavior, and antimicrobial resistance rates at community level influence on transmission of resistant microorganisms. The transmission of commensal bacteria between individuals and antibiotic use occur all the time, this ensures selection of resistant bacteria in the community [55]. Uganda being a low resource country, several factors are likely to be playing a role in the transmission of these resistance traits. The findings from rural central Uganda show that families with poor hygienic practices and environmental hygiene were significantly associated with having helminthes transmitted by oral faecal spread, which would equally have transmission of resistant bacteria [56]. The sanitation standards in the urban areas are worsened by low levels of funding and supervision of sewage systems with frequent over flow of sewerage onto the surface [57]. On the other hand 70 % of the urban poor use shared latrines that quite often fail to serve the desired need of improved sanitation [58]. Furthermore there are unhygienic practice by food vendors in the region covering the study area [59]. In a low resource setting purchase transaction involves use of bank notes and yet in the recent past contaminated bank notes have been identified as contributing to the transmission of ESBLs [60]. It is possible this occurs among individuals who may have no history of using antibacterial agents in this setting too.

The level of resistance noted in this study has several implications. With minimal laboratory support for culture and sensitivity the chances of ineffective chemotherapy, treatment failure and increased duration of hospital stay and death are likely to be rampant [61]. With prolonged illness, there is increased risk of spread of infection to other people with ultimate increase in costs of health care [62]. Uganda being a low income country, treatment of multidrug resistant bacteria necessitating the purchase of expensive last line antimicrobials is not easy to achieve in the public facilities [63, 64]. Thus these findings call for initiation of efforts to continuously collect susceptibility data from both rural and urban areas to inform the planning of antibiotic policies and introducing antibiotic stewardship programs at all levels of health care delivery.

One limitation in this study was that we considered three colonies from all specimen with successful growth, instead of using antibiotic containing media to screen for antimicrobial resistance phenotypes. This could have led to missing some ESBLs. However, the study gives insight into the problem of MDR among ESBL negative bacteria. Use of client prescription notebooks and recall to give information about antimicrobials previously taken, may have left some antimicrobial use undetected. The use of specimen as opposed to rectal swabs affected the yield of flora. The findings from this study are based on clients attending outpatient clinics in the selected districts of central Uganda and may not be generalized to clients attending outpatient clinics throughout the country.

## Conclusions

Antimicrobial resistance is wide-spread among *E. coli* and *K. pneumoniae* in clients attending outpatient clinics in Kampala and rural districts of Uganda. This could complicate treatment options for community acquired infections caused by the enterobacteriaceae.

## Additional files

10.1186/s13104-016-2049-8 Sample questionnaire showing the data capture tool used at Outpatient clinics.
10.1186/s13104-016-2049-8 This additional table shows numbers of outcomes of events in relation to the exposure(s). It complements number 15 of the STROBE guideline checklist.
10.1186/s13104-016-2049-8 The STROBE guidelines checklist.

## Abbreviations

AmpC: class C beta-lactamase enzyme (cephalosporinase)
ESBL: extended spectrum beta-lacatamase enzyme
MDR: multidrug resistant
HIV/AIDS: human immunodeficiency virus/aquired immunodeficiency syndorme
HC: health center
HSD: health sub-district
PNFP: private not for profit
HPI: human poverty index
CI: confidence interval
ISS: immunosuppresive syndrome
CAP: College of American pathologists
CLSI: clinical laboratory standards institute
DST: drug susceptibility testing
MHA: Mueller–hinton agar
SIN: sulphide indo-motility agar
TSI: tripple sugra and iron
ATCC: American type culture collection
MIC: minimum inhibitory concentraion
DDST: double disk synergy test
SD: standard deviation
IRB: institutional review board
cOR: crude odds ratio
aOR: adjusted odds ratio
BCP: basic care package
SIDA: Swedish International Development Cooperation Agency
