# Supplementary material for: Antimicrobial susceptibility profiles of Escherichia coli and Klebsiella pneumoniae isolated from outpatients in urban and rural districts of Uganda
Source: BMC Res Notes. 2016 Apr 25;9:235. doi: 10.1186/s13104-016-2049-8 (PMC4843195; doi:10.1186/s13104-016-2049-8)
Supplement: Supplementary file 1 — 10.1186/s13104-016-2049-8 Sample questionnaire showing the data capture tool used at Outpatient clinics. [file 13104_2016_2049_MOESM1_ESM.pdf]

FORM A: (*questionnaire*)

Form No……………………

ESBL STUDY

FIELD DATA COLLECTION FORM

ID NO……………………………………………………………………………………………….

DATE………………………………………………………………………………………………

Age……………………………………………………………………………………….

Sex………………………………………………………………………………………………

Parish……………………………………………………………………………………………..

Subcounty……………………………………………………………………………………..

OPD……………………………………………………………………………………………...

HC………………………………………………………………………………………………..

HSD……………………………………………………………………………………………..

District…………………………………………………………………………………….

Reason for hospital

Visit………

H/O treatment (recent past)

Duration	Drug(1)	Drug(2)	Drug(3)	Drug(4)
Current week				
1 week ago				
2 weeks ago				
3 weeks ago				
4 weeks ago				
5 weeks ago				
6 weeks ago				
7 weeks ago				
8 weeks ago				

(If patient treated more than 8 to 12 weeks ago indicate drug at bottom of the table)

……….

History of admission in the last 3 month? Y/N (tick)

History of medical procedure last 3 month? (*Some description*) 4-5

Words)……

Signature……………………………………………………………print Name………………………………………………………………
