# Supplementary material for: Antimicrobial susceptibility profiles of Escherichia coli and Klebsiella pneumoniae isolated from outpatients in urban and rural districts of Uganda
Source: BMC Res Notes. 2016 Apr 25;9:235. doi: 10.1186/s13104-016-2049-8 (PMC4843195; doi:10.1186/s13104-016-2049-8)
Supplement: Supplementary file 2 — 10.1186/s13104-016-2049-8 This additional table shows numbers of outcomes of events in relation to the exposure(s). It complements number 15 of the STROBE guideline checklist. [file 13104_2016_2049_MOESM2_ESM.pdf]

Table S1: Numbers of Outcome variables by predictor variable

	ESBL (total 52)		AmpC (total (129)		MDR (total 355)	
	n/N (%)	P-value	n/N (%)	P-value	n/N (%)	P-value
**Characteristics**						
**age**						
0-14	15/21 (71%)	0.270	37/61 (61%)	0.259	90/310 (29%)	[a] 0.004**
15-44	31/36 (86%)		79/158 (50%)		217/538(40%)	
45+	6/6 (100%)		12/27(44%)		48/133(36%)	
**Sex**						
Female	30/38 (79%)	0.503	83/170 (49%)	0.132	244/649 (38%)	0.188
Male	22/25 (88%)		45/76 (59%)		111/333 (33%)	
**HC level**						
NR	17/23 (74%)	[a]0.037**	26/45 (58%)	0.846	68/103(66%)	[a] 0.001**
GH	14/17 (82%)		39/77(51%)		104/271(38%)	
HCIV	3/5 (60%)		13/27(48%)		53/153(35%)	
HCIII	18/18 (100%)		51/98(52%)		131/458 (29%)	
**District**						
Kampala	35/42 (83%)	0.886	65/122(53%)	0.593	178/274 (78%)	[a] 0.001**
Kayunga	7/9 (78%)		27/58(47%)		97/406 (24%)	
Mpigi	10/12 (83%)		37/67 (55%)		81/305 (27%)	
**HSD**						
Cases>100	31/41 (76%)	0.079**	69/132 (52%)	0.988	203/641(32%)	[a] 0.001**
Cases<100	21/22(95%)		60/115 (52%)		153/342 (48%)	
**Reason For visit**						
ISS	5/7 (71%)	0.426	5/14 (36%)	[a] 0.003**	27/87 (31%)	0.441
Infection	28/34 (82%)		60/138 (43%)		186/488 (38%)	
General	19/21 (90%)		57/87 (66%)		132/363 (36%)	
**History of admission**						
Yes	7/7 (100%)	0.337	19/26 (73%)	[a] 0.023**	33/78 (42%)	0.274
	45/56		109/220		323/895	

			(50%)		(36%)	
**HistoryOf Medical procedures**						
contact	1/1 (100%)	n/a	0/4 (0%)	[a] 0.044**	1/6 (17%)	0.075**
Inoculation	5/5 (100%)		7/17 (41%)		23/51 (45%)	
Surgery	1/1 (100%)		3/3 (100%)		2/14 (14%)	
**Antibiotic use**						
Yes	41/46 (89%)	[a] 0.023**	96/190 (51%)	0.329	263/755 (35%)	0.122
No	11/17 (65%)		33/57 (58%)		93/230 (40%)	
Use of gentamicin						
Yes	½ (50%)	1.000	2/9 (22%)	0.100	10/27 (37%)	0.806
No	40/45(89%)		94/182 (52%)		254/731 (35%)	
Use of ciprofloxacin						
Yes	8/10 (80%)	0.306	15/28 (54%)	0.682	37/85 (44%)	0.071**
No	32/35 (91%)		80/162 (49%)		226/672 (34%)	
Use of septrin						
Yes	26/28 (93%)	0.088**	68/130 (52%)	0.858	175/543(32%)	[a] 0.001**
No	24/32 (75%)		47/92 (51%)		141/299 (47%)	
Use of penicillins						
Yes	7/8(88%)	0.566	32/61 (52%)	0.605	88/244 (36%)	0.574
No	33/36 (92%)		62/128 (48%)		174/512 (34%)	
Use of tetracycline						
Yes	1/1(100%)	1.000	0/2 (0%)	0.236	5/24 (21%)	0.192
No	44/50 (88%)		100/193 (52%)		267/751 (36%)	
Use of chloramphenicol						
Yes	3/3(100%)	1.000	3/8 (38%)	0.493	15/34 (44%)	0.258
No	38/44(86%)		96/187 (51%)		254/733 (35%)	
Use of ceftriaxone						
Yes	1/1(100%)	1.000	½ (50%)	1.000	3/8 (38%)	1.000
No	44/50(88%)		99/193 (51%)		269/767 (35%)	
Use of Erythromycin						
Yes	2/2(100%)	1.000	9/16 (56%)	0.647	14/54 (26%)	0.153
No	39/45 (87%)		89/177 (50%)		252/709 (36%)	

NR, national referral; GH, General hospital; HCIV, health center 4; HCIII, health center III; HSD, health sub-district; ISS, immune suppression syndrome (HIV/AIDS); ref, used as reference (district, health center level, reason for visit); n/a, not applicable (none of the exposed had esbl);[a] significant association.

** used in the model
