# Supplementary material for: Antimicrobial susceptibility profiles of Escherichia coli and Klebsiella pneumoniae isolated from outpatients in urban and rural districts of Uganda
Source: BMC Res Notes. 2016 Apr 25;9:235. doi: 10.1186/s13104-016-2049-8 (PMC4843195; doi:10.1186/s13104-016-2049-8)
Supplement: Supplementary file 3 — 10.1186/s13104-016-2049-8 The STROBE guidelines checklist. [file 13104_2016_2049_MOESM3_ESM.pdf]

STROBE Statement Checklist of items that should be included in reports of cross-sectional studies

	Item No.	Recommendation	Page
**Title and abstract**	1	(*a*) Indicate the study's design with a commonly used term in the title or the abstract	3
		(*b*) Provide in the abstract an informative and balanced summary of what was done and what was found	3-4
**Introduction**			
Background/rationale	2	Explain the scientific background and rationale for the investigation being reported	5-6
Objectives	3	State specific objectives, including any prespecified hypotheses	6
**Methods**			
Study design	4	Present key elements of study design early in the paper	6
Setting	5	Describe the setting, locations, and relevant dates, including periods of recruitment, exposure, follow-up, and data collection	6-7<br>8-11
Participants	6	(*a*) Give the eligibility criteria, and the sources and methods of selection of Participants	7<br>8
Variables	7	Clearly define all outcomes, exposures, predictors, potential confounders, and effect modifiers.	11
		Give diagnostic criteria, if applicable	8
Data sources/	8*	For each variable of interest, give sources of data and details of methods of assessment (measurement).	8-11
measurement		Describe comparability of assessment methods if there is	
		more than one group	
Bias	9	Describe any efforts to address potential sources of bias	8<br>8-11
Study size	10	Explain how the study size was arrived at	7-8
Quantitative	11	Explain how quantitative variables were handled in the analyses. If applicable, describe;	12

variables			
		Which groupings were chosen and why	NA
Statistical methods	12	(*a*) Describe all statistical methods, including those used to control for confounding	11-12
		(*b*) Describe any methods used to examine subgroups and interactions	NA
		(*c*) Explain how missing data were addressed	A
		(*d*) If applicable, describe analytical methods taking account of sampling strategy	11
		(*e*) Describe any sensitivity analyses	NA
**Results**			
Participants	13*	(a) Report numbers of individuals at each stage of study—e.g. numbers potentially eligible, examined for eligibility, confirmed eligible, included in the study, completing follow-up, and analyzed	
		(b) Give reasons for non-participation at each stage	
		(c) Consider use of a flow diagram	Fig.2.
Descriptive data	14*	(a) Give characteristics of study participants (e.g. demographic, clinical, social) and information on exposures and potential confounders	12-13
		(b) Indicate number of participants with missing data for each variable of interest	
Outcome data	15*	Report numbers of outcome events or summary measures	Table S1
Main results	16	(*a*) Give unadjusted estimates and, if applicable, confounder-adjusted estimates and their precision (e.g. 95% confidence interval). Make clear which confounders were adjusted for and why they were included	Table 4
		(*b*) Report category boundaries when continuous variables were categorized	NA
		(*c*) If relevant, consider translating estimates of relative risk into absolute risk for a meaningful time period	NA
Other analyses	17	Report other analyses done e.g. analyses of subgroups and interactions, and sensitivity analyses	NA
**Discussion**			
Key results	18	Summarize key results with reference to study objectives	18-21
Limitations	19	Discuss limitations of the study, taking into account sources of potential bias or imprecision.	25

		Discuss both direction and magnitude of any potential bias	
Interpretation	20	Give a cautious overall interpretation of results considering objectives, limitations, multiplicity of analyses, results from similar studies, and other relevant evidence	
Generalizability	21	Discuss the generalizability (external validity) of the study results	25
**Other information**			
Funding	22	Give the source of funding and the role of the funders for the present study and, if applicable, for the original study on which the present article is based	28

a = Summary tabulation of each of the variables was done to identify the extent of missing variables. This was less than 5% hence no variable was excluded from the analysis due to missing data
